# The Intriguing Links between Psoriasis and Bullous Pemphigoid

**DOI:** 10.3390/jcm12010328

**Published:** 2022-12-31

**Authors:** Carlo Alberto Maronese, Nicoletta Cassano, Giovanni Genovese, Caterina Foti, Gino Antonio Vena, Angelo Valerio Marzano

**Affiliations:** 1 Dermatology Unit, Fondazione IRCCS Ca’ Granda Ospedale Maggiore Policlinico, 20122 Milan, Italy; 2 Department of Pathophysiology and Transplantation, Università degli Studi di Milano, 20122 Milan, Italy; 3 Dermatology Private Practice, 76121 Barletta, Italy; 4 Dermatology Private Practice, 70125 Bari, Italy; 5 Department of Precision and Regenerative Medicine and Ionian Area, Unit of Dermatology, University of Bari Aldo Moro, 70124 Bari, Italy

**Keywords:** psoriasis, bullous pemphigoid

## Abstract

The coexistence of psoriasis with autoimmune bullous diseases (AIBDs), particularly bullous pemphigoid (BP), has been documented in case reports and series, as well as in epidemiological studies. The onset of psoriasis precedes that of BP in the majority of cases. Patients with concomitant BP and psoriasis are generally younger at the onset of BP and present with fewer erosions and blisters as compared with patients suffering from isolated BP. Intriguingly, it has been speculated that some BP cases with comorbid psoriasis can actually correspond to anti-laminin gamma-1 pemphigoid, a rare form that was recently recognized as a distinct entity and which can mimic BP and/or other subepidermal AIBDs. The pathomechanisms underlying the BP–psoriasis association have not yet been identified, although several hypotheses have been proposed. The most credited among such hypotheses involves the so-called “epitope spreading” phenomenon, with tissue injury secondary to a primary inflammatory process (i.e., psoriasis) leading to the exposure of sequestered antigens evoking a secondary autoimmune disease (i.e., bullous pemphigoid). This narrative review aims to give a brief overview of the association between psoriasis and BP, examining epidemiological, clinical, and immunopathological features, the pathomechanisms underlying this association, the treatments for psoriasis incriminated as potential triggers of BP, and the therapeutic management of patients with psoriasis and BP.

## 1. Introduction 

Psoriasis is a rather common chronic inflammatory skin disease, resulting from a complex interplay between genetic, environmental, and immunological factors. The most frequent clinical variant is chronic plaque psoriasis (psoriasis vulgaris), characterized by erythematous plaques with well-demarcated borders covered by silvery-white scales. It has a bimodal incidence, manifesting both in young adults and middle-aged subjects, although it may appear at any age [1].

Conversely, autoimmune blistering diseases (AIBDs) are a heterogeneous group of disorders mediated by circulating autoantibodies against antigens of skin and mucous membrane components. Bullous pemphigoid (BP) represents the most common subepidermal AIBD and typically affects elderly people [2]. 

BP is typified by the presence of tense blisters on either erythematous or normal-appearing skin, although atypical presentations with urticarial, papular or eczema-like eruptions have been described. Pruritus is common and mucosal lesions, generally limited to the oral cavity, are present in up to 30% of patients [3]. BP is linked to the production of autoantibodies against BP180 (type XVII collagen) and BP230, two structural components of the basement membrane zone (BMZ). BP180-specific autoantibodies appear to play a primary role in the pathogenesis of BP, and the majority of BP patients have autoantibodies targeting the immunogenic extracellular noncollagenous 16A domain (NC16A) of BP180 [4].

The coexistence of psoriasis with AIBDs has been documented in case reports and series, as well as in epidemiological studies [5,6].

The aim of this narrative review is to provide a brief overview of the association between psoriasis and BP, examining the epidemiological, clinical, and immunopathological aspects, the pathomechanisms underlying this association, the treatments for psoriasis implicated as potential triggers of BP, and the therapeutic management of patients suffering from both conditions. Our review did not focus on pustular psoriasis, which has distinct clinical, histological, and pathophysiological features compared to psoriasis vulgaris [7].

For our purposes, electronic searches were performed on the PubMed database and Google Scholar using the keywords “psoriasis” and “bullous pemphigoid.” Articles in English, published up to 31 October 2022, were selected, and full copies of eligible articles, including case reports and review articles, were collected. The references of retrieved manuscripts were also checked to find additional eligible articles.

## 2. Epidemiological Data

Various studies have highlighted the association between psoriasis and AIBDs.

In a retrospective evaluation of 145 Japanese patients with both psoriasis and AIBDs performed by Ohata et al. [5], psoriasis vulgaris was documented in 84.1% of cases and pustular psoriasis in 9%, while BP was the most common AIBD (63.4%), followed by anti-laminin gamma-1 (p200) pemphigoid (37.2%). The authors speculated that patients with pustular psoriasis may be at a higher risk of developing AIBDs, with anti-laminin gamma-1 pemphigoid being diagnosed in 53.8% of subjects with pustular psoriasis in their series [5].

An assessment of comorbidities in psoriasis patients using a national database in Taiwan revealed a significantly increased prevalence of BP (relative ratio 14.75, 95% confidence interval (CI) 5.00–43.50) [8]. In a subsequent nationwide population-based cohort study in Taiwan recruiting 109,777 psoriatic patients and 109,777 controls, psoriasis was significantly associated with an increased risk of BP (hazard ratio 3.05, 95% CI 2.10–4.43) [9]. BP was diagnosed after a mean period of 2.86 years following the diagnosis of psoriasis. In this study [9], psoriatic patients with BP were more likely to have a psychiatric disease. 

Donnelly et al. reviewed a total of 76 cases of psoriasis and concurrent BP, retrieved by a literature search up to June 2015, using the PubMed and SciVerse Scopus databases [10]. In such cases, the male-to-female ratio was 2.3:1, the average age of disease onset was 45 years (standard deviation (SD) 20 years) for psoriasis and 65 years (SD 12 years) for BP. Psoriasis preceded BP in all cases but one, and the mean interval between diagnoses of the two diseases was 21 ± 17 years. Unsurprisingly, the most common variant of psoriasis was plaque psoriasis, accounting for 79% of patients. 

The significant association between BP and psoriasis was confirmed by a meta-analysis of case-control studies published up to March 2018, involving 4035 adult patients with BP and 19,215 controls [11]. In detail, the prevalence of psoriasis was significantly higher in BP patients compared to controls (2.6% vs. 1.1%, odds ratio (OR) 2.5, 95% CI 1.4–4.6). The association was significant in both males and females, but significantly more cases were found among the former.

A study from Israel evaluated the bidirectional association between BP and psoriasis [12]. Using a case-control design, preexisting psoriasis was found to be significantly more frequent among patients with BP relative to the controls (OR 1.53, 95% CI 1.17–2.02). Furthermore, a population-based retrospective cohort study assessed incident cases of psoriasis in 3924 BP patients and 19,280 age-, sex-, and ethnicity-matched controls. The adjusted risk of incident psoriasis was 2.6-fold higher among BP patients compared to the controls. In patients with BP and psoriasis, BP followed psoriasis in 67.3% of cases (mean latency 11.8 years), while it preceded the diagnosis of psoriasis in the remaining 32.7% (mean interval 3.8 years). There were no differences between patients with concurrent BP and psoriasis and those with BP alone in terms of ethnicity, average body mass index, prevalence of diabetes mellitus or dyslipidemia, and average Charlson comorbidity score. Moreover, the prevalence of gliptin- or anti-PD-1/PDL-1-associated BP was superimposable in the two groups. Patients with concomitant BP and psoriasis were more likely to be male, smokers, and hypertensive as compared to those suffering from BP alone [12].

It should be mentioned that a female predominance has been observed in most studies on classic BP, in contrast with the higher prevalence of males among patients with both psoriasis and BP [10,11,12,13].

Another interesting finding that consistently emerged throughout the literature is that patients with concomitant BP and psoriasis are significantly younger at BP onset as compared with patients with isolated BP [9,10,12,14].

## 3. Clinical and Immunopathological Features

Comparisons between patients with BP and concurrent psoriasis and patients with BP alone revealed no significant differences in terms of the anatomical distribution of the bullous lesions [14,15], whereas the frequency of mucosal involvement was reported by some authors to be similar in the two groups [15] or, in another report, marginally higher among BP patients with coexisting psoriasis [14].

There are reports of patients with psoriatic erythroderma and BP [16,17,18,19,20], presenting as pemphigoid nodularis in one case [21]. In a patient with BP and erythrodermic psoriasis, the authors emphasized the absence of any apparent relationship to antipsoriatic systemic or topical treatments [20].

In one patient, BP was seen soon after a pustular flare of psoriasis, as a sign of an active hyperinflammatory condition following the sudden interruption of a potent topical corticosteroid [22].

Interestingly, a flare of psoriasis immediately before BP development was documented in 38% of the cases reviewed by Donnelly et al. [10], manifesting as an erythrodermic eruption in 16% of cases.

**Figure 1:**
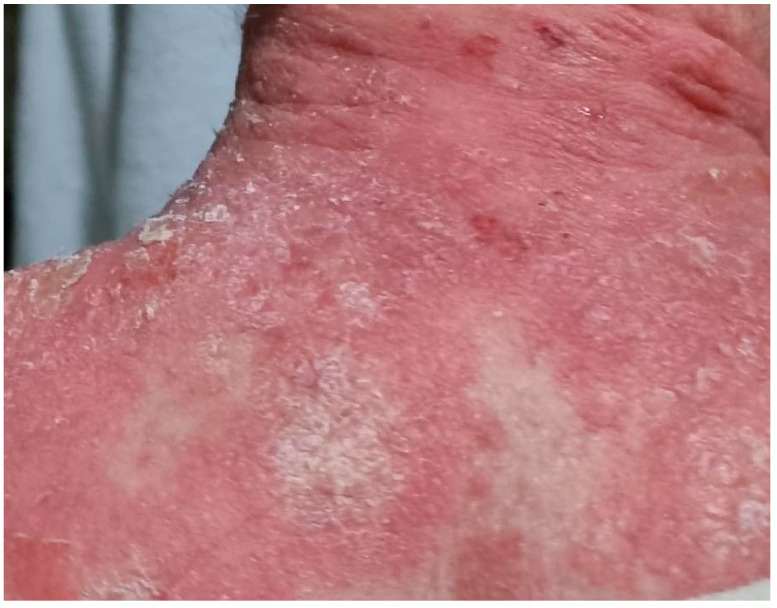
Clinical features of a patient with concurrent psoriasis and bullous pemphigoid, showing erosive lesions in the context of psoriatic plaques.

In some individuals with a dual diagnosis of BP and psoriasis, colocalization of bullous and psoriatic lesions has been observed [5,10,15], as shown in Figure 1, and bullous lesions limited to psoriatic plaques were also described [23].

BP patients with comorbid psoriasis were found to have fewer erosions/blisters, while there were no differences in the severity of the erythematous component or pruritus, nor were there differences regarding the frequency of positive anti-BP180 NC16A serum autoantibodies [15].

Nevertheless, Ständer et al. noted that subjects suffering from BP and comorbid psoriasis had lower levels of anti-BP180 NC16A autoantibodies, possibly reflecting the milder erosive phenotype, and also a higher frequency of isolated linear C3 deposits and a lower frequency of linear IgG deposits along the BMZ, detected by direct immunofluorescence microscopy [15]. 

Moreover, cases of BP with concurrent psoriasis can display atypical immunopathological features, such as seronegativity for anti-BP180 NC16A antibodies or neutrophil-predominant inflammatory infiltrates [24,25]. 

The coexistence of psoriasis and BP in association with other diseases, such as Hashimoto’s thyroiditis, metabolic syndrome, breast cancer with Parkinson’s disease, vitiligo, sarcoidosis, and macroglobulinemia has been observed [26,27,28,29,30,31,32].

It is possible that some BP cases with comorbid psoriasis found in the literature could actually correspond to anti-laminin gamma-1 pemphigoid, as this rare form, recently recognized as a distinct entity, can mimic BP and other subepidermal AIBDs. Anti-laminin gamma-1 (p200) autoantibodies were tested sporadically in the reported cases [10], considering that specific detective technology is not available in most countries. Indeed, psoriasis has been reported in nearly 28% of anti-p200 pemphigoid patients, with higher rates among Japanese patients, who also seem to display a stronger association between anti-p200 pemphigoid and pustular psoriasis [5,33]. 

## 4. Pathogenetic Insights

### 4.1. Psoriasis

A central role in the pathogenesis of psoriasis vulgaris has been ascribed to the proinflammatory cytokines tumor necrosis factor (TNF)-alpha, interleukin (IL)-23, and IL-17, with upregulation of the Th1 and Th17 subsets and dysfunction of the regulatory T cells [34,35,36,37,38].

Both the innate and adaptive immune responses are involved in psoriatic inflammation. In the early stages, antimicrobial peptides and antigenic stimuli activate plasmacytoid dendritic cells to produce interferon (IFN)-alpha, promoting the activation of myeloid dendritic cells. In the maintenance phase, the TNF-alpha/IL-23/IL-17 axis plays a crucial role in the vicious inflammatory loop, and IL-23 has a fundamental role in stimulating the production of IL-17 [38,39,40,41].

Moreover, erythrodermic psoriasis appears to also be characterized by an increased Th2 response, the actual contribution of which needs to be clarified [42,43], and by the involvement of the IL-17 pathway [44]. 

The association of psoriasis with autoimmune disorders and the recognition of autoreactive T cells as pathogenetic contributors hint at a possible autoimmune component in psoriasis pathogenesis [45,46,47].

### 4.2. Bullous Pemphigoid 

Th2 cells are recognized as the primary drivers of antibody production in BP, although the precise pathogenetic processes are not completely known, and various data support the involvement of other components, such as Th17 cells, in the exacerbation of the inflammatory response, and regulatory T cells, whose dysregulation promotes the activation of autoreactive T cells and autoantibody production [48,49,50].

Complement activation, mast cell degranulation, the accumulation of inflammatory cells, particularly eosinophils, which are the predominant cells in the inflammatory infiltrate, along with mast cells and neutrophils, and the release of proteases from inflammatory cells are included among the pathophysiological events leading to dermal-epidermal detachment in BP [49,51,52].

The role of several cytokines, including IL-1beta, IL-4, IL-5, IL-6, IL-8, IL-18, IL-31, IFN-gamma, and TNF-alpha, along with chemokines, has been suggested [53,54,55,56]. A meta-analysis showed that BP patients have significantly increased serum concentrations of IL-5, IL-6, IL-8, IL-17, CCL17, and CCL26, and increased blister fluid levels of IL-5, IL-6, IL-8, CCL11, and TNF-alpha [57]. 

IL-17 and IL-23 were found to upregulate the expression of proteases involved in blister formation and cleavage of the extracellular domain of BP180; elevated serum levels of these two cytokines can have a prognostic value, helping to identify BP patients at risk of future relapse [58,59]. IL-17 and IL-23 were also identified as essential molecules favoring the expression of IL-1beta in macrophages from BP patients, with IL-1beta driving inflammasome activation [60]. 

The relevance of IL-17A in BP pathogenesis has been suggested by additional findings, including the upregulation of cytokine and related genes in the skin of BP patients, the ability of IL-17A to activate neutrophils, and evidence of BP in animal models [61]. 

Moreover, in BP, IL-23 promotes the formation of neutrophil-derived DNA extracellular traps, which are well-known to participate in the loss of tolerance processes of several autoimmune diseases [62].

### 4.3. Bullous Pemphigoid–Psoriasis Association 

The pathomechanisms underlying BP–psoriasis association have not yet been identified, although several hypotheses have been proposed, and a multifactorial nature cannot be ruled out. Some hypotheses have taken into account the pathophysiological features of psoriasis, with plausible implications in BP pathogenesis, as psoriasis preceded BP in the majority of reported cases. However, the exact reasons for the switch from a Th1/Th17- to a Th2-dominant cytokine milieu are still unknown [63]. In patients with psoriatic erythroderma, a shift towards a Th2 profile can occur, contributing to BP development [10]. Nevertheless, in some cases of concurrent BP and psoriasis, BP preceded the diagnosis of psoriasis. 

Psoriasis comorbidities and treatments used to manage such comorbidities, and/or psoriasis itself, have been incriminated in the development of BP, in some cases [11]. Among treatments for psoriasis, ultraviolet (UV) irradiation has often been described as a trigger of BP and other AIBDs and might be responsible for the exposure or release of altered BMZ antigens [5].

The contributory role of infectious agents has been speculated in both BP and psoriasis, as well as in triggering immunological responses against BMZ components which may have been altered by the inflammatory processes of psoriasis or its treatment [64].

Pathological events at the BMZ and epigenetic changes in psoriatic skin can precipitate AIBDs in genetically predisposed subjects, probably by altering, unveiling, or exposing BMZ antigens. Increased epithelial turnover, with persistent inflammatory changes, active recruitment of abundant immune cells, and degradation of BMZ components, might modify the BMZ antigenicity and promote antigen exposure to autoreactive T cells and autoantibody production [6,11,63]. 

The presence of the two diseases was attributed to the so called “epitope spreading” phenomenon. In essence, tissue injury secondary to a primary inflammatory process allows the exposure of sequestered antigens, evoking a secondary autoimmune disease [65]. In a case series of patients with psoriasis and AIBDs, epitope spreading resulting from BMZ damage was also recognized as a possible explanation for the development of non-pathogenic autoantibodies in the absence of related clinical manifestations [5].

The loss of BMZ integrity, possibly resulting from the activity of proteolytic enzymes, has been described in psoriasis, and such changes may unmask antigenic sites in the BMZ [11,12,23]. Neutrophil elastase, identified along the BMZ of psoriatic plaques, could have a role in BMZ disruption [66].

Data have suggested the existence of alterations, even in psoriatic non-lesional skin, such as the overexpression of matrix metalloproteinases and the disruption of the laminin layer within the BMZ [67,68]. Therefore, even in the psoriatic non-lesional tissue, abnormalities in the BMZ proteins may lead to the exposure of novel epitopes [68].

According to another hypothesis, senescence contributes to alter BMZ antigenicity, favoring autoimmune responses against BMZ components. It was suggested that the extracellular matrix in psoriatic skin can simulate the senescent extracellular matrix, increasing the risk of the development of BP and other AIBDs [6].

Additionally, a shared pathogenetic role of proinflammatory cytokines, such as IL-1, IL-17, and IL-23, as well the role of neutrophils, responsible for the degradation of matrix proteins, have been hypothesized in both psoriasis and BP [12]. The possible involvement of IL-31 in BP-associated pruritus has been proposed, with evidence of elevated levels in BP lesional skin and blister fluid, whereas the role of this pruritogenic cytokine in psoriasis is still elusive [55,69]. Recent evidence has shown elevated serum levels of IL-31 in psoriasis patients, with distinct IL-31 promoter gene polymorphisms possibly involved in psoriasis pathogenesis. However, no correlation between itch or disease severity and IL-31 serum levels has been detected [69].

#### Treatments for Psoriasis as Triggers of BP

It has been reported that BP and other AIBDs may be induced or exacerbated by antipsoriatic treatments, such as coal tar and phototherapy [10,70,71].

In 58% of the cases reviewed by Donnelly et al., treatments for psoriasis were credited as triggers of BP, with UV light therapy, especially oral psoralen with UVA (PUVA), as the most cited treatment associated with BP onset [10].

UV radiation might induce injury in the basal cells, conformational changes in BP antigen, or modifications of BMZ antigenicity, with possible exposure or release of modified antigens and stimulation of autoreactive T cells or autoantibody formation [5,65,72]. Some authors hypothesized that both phototherapy and a higher severity of psoriasis influence BMZ antigenicity [9]. Moreover, PUVA therapy is capable of shifting cytokine expression from Th1 to Th2 [72]. Sugita et al. noted the occurrence of BP, combined with the detection of increased circulating Th2 cells, in a patient when his psoriasis was successfully controlled by PUVA therapy, along with the subsequent recurrence of psoriasis in parallel with BP improvement and normalization of Th2 deviation [73]. 

According to a systematic review published in 2020 by Verheyden et al. [74], among medications implicated in drug-induced BP, the association was reported as likely for PUVA and uncertain for dithranol and coal tar. 

An eruption of BP within psoriatic plaques following cyclosporine cessation has been described [75].

Interestingly, AIBD onset during treatment of chronic inflammatory diseases (rheumatoid arthritis, ulcerative colitis, Crohn’s disease, hidradenitis suppurativa, and psoriasis) with biologics has been observed [76,77,78]. TNF-alpha inhibitors were the most frequently implicated drugs, and psoriasis was the most common condition for which the biologic drug was prescribed [77]. In the majority of patients, the AIBD resolved after the interruption of the biological agent, and in patients who underwent re-treatment, the AIBD often relapsed [77].

Tirado-Sánchez et al. described a patient who developed BP and vitiligo after receiving treatment with adalimumab for psoriasis [79]. 

Verheyden et al., in their review regarding drug-induced BP74, proposed a probable association with monoclonal antibodies targeting TNF-alpha (adalimumab, infliximab), the p40 subunit of IL-12 and IL-23 (ustekinumab), and the CD11a subunit of the lymphocyte function-associated antigen-1 (efalizumab, withdrawn from the market in 2009), whereas the association with another TNF inhibitor, the p75 TNF receptor-Fc fusion protein etanercept, was considered likely [74].

Curiously, biological agents implicated as causative factors of AIBDs were successfully used for the treatment of a few patients with AIBDs [80,81,82,83,84,85,86]. No clear explanations exist for these paradoxical findings. For instance, TNF-alpha seems to play a relevant role in the pathogenesis of BP [77,87]. However, treatment with TNF inhibitors has been associated with aggravation of pre-existing autoimmune diseases and the onset of new inflammatory diseases and autoimmune phenomena [88]. Neutralization or depletion of TNF-alpha could enhance autoreactive B cells and humoral autoimmunity, as well as the production of IFΝ-alpha by plasmacytoid dendritic cells [77]. Anti-TNF-alpha agents have been hypothesized to display opposing effects on autoimmune disorders, having the ability to induce or treat such disorders, depending on the immunological profile and levels of IL-4 and IFN-gamma [76].

A recent systematic review identified 15 case reports of BP during treatment with biologics for psoriasis, and the correlation was rated as probable in the majority of cases [89]. In particular, the culprit drugs were ustekinumab in six patients (all with a previous failure of anti-TNF biological therapy), efalizumab in three, etanercept in three, adalimumab in two, and secukinumab in one case. The mean period of latency until BP developed was different between TNF-alpha inhibitors and ustekinumab (5.12 ± 3.44 weeks and 28.66 ± 26.27 weeks, respectively), possibly suggesting distinct pathomechanisms. 

Drugs inhibiting the IL-23/IL-17 pathways were suggested as potential treatment approaches for severe refractory BP [85]. The IL-12/IL-23 blocker ustekinumab was effective in a patient with BP and psoriasis [85], but paradoxical reactions are possible, as suggested by cases of BP arising in psoriatic patients receiving ustekinumab [89].

The occurrence of BP during treatment of psoriasis with IL-17 inhibitors and anti-IL-23p19 agents was rarely described and seems paradoxical, as IL-17 and IL-23 are overexpressed in BP [59,90,91,92]. In the case report written by Ho et al. [90], the strength of the causal relationship between BP and the use of the IL-17 inhibitor secukinumab was weakened by the absence of BP recurrence after re-treatment with secukinumab. The psoriatic patients with the onset of BP during therapy with the IL-23 blockers guselkumab and risankizumab were elderly, and the patient treated with guselkumab had bullous lesions appearing 4 weeks after switching from ustekinumab [91,92]. 

In the opinion of Husein-ElAhmed et al. [89], the development of BP during treatment with biologics might result from the interference with the immune balance, rather than from a specific effect on the cytokine pathways.

## 5. Management of Concurrent Psoriasis and Bullous Pemphigoid

Treatment strategies for psoriasis or BP should take into consideration several factors, including disease severity and comorbidities. 

Topical agents, especially corticosteroids, vitamin D3 analogues, keratolytics, and combinations of these, represent the mainstays of therapy for mild psoriasis. More severe forms of psoriasis may require phototherapy or photochemotherapy (PUVA) and systemic medications (e.g., methotrexate, cyclosporin, acitretin, apremilast, fumarates, and biologics). Biologic therapies currently approved for moderate-to-severe psoriasis includes agents targeting TNF-alpha, IL-17, the p40 subunit of IL-12 and IL-23, and the p19 subunit of IL-23 [38].

The treatment of BP is largely based on immunosuppressants, with systemic glucocorticoids being the mainstay of treatment. Other effective medications are superpotent topical steroids, tetracyclines, alone or combined with nicotinamide, and dapsone, while immunosuppressants (i.e., azathioprine, methotrexate, cyclosporin, mycophenolates) are usually added to oral corticotherapy due to their steroid-sparing effect. Other options that can be considered for selected severe refractory cases include intravenous immunoglobulin, plasma exchange, immunoadsorption, the anti-CD20 monoclonal antibody rituximab, the anti-IgE monoclonal antibody omalizumab, and dupilumab, a monoclonal antibody targeting the IL-4 receptor alpha chain [93]. The available data regarding BP patients, obtained from small uncontrolled studies or single reports, appear to indicate similar effectiveness of the above-mentioned monoclonal antibodies, whose use in BP is, however, off-label and not yet validated [93,94]. 

Clinical trials in BP patients have recently evaluated or are still assessing the activity of dupilumab, rituximab, alone or combined with omalizumab, and other therapeutic approaches, including the blockade of IL-17A, IL-12/IL-23, and IL-23 [95].

Definite conclusions about the optimal management of patients suffering from BP and psoriasis cannot be drawn, as the available evidence mainly consists of retrospective analyses of small case series or case reports with a limited number of total patients. 

An important aspect in the management of cases with suspected triggers is the discontinuation of culprit agents.

Thus, treatment of patients with coexistent BP and psoriasis can be challenging. 

Therapies for psoriasis, such as phototherapy and certain biologics [10,89], as specified in the previous paragraph, have been reported to trigger BP. Conversely, the onset of psoriasis has been described during treatment with rituximab or dupilumab [96,97,98].

Tetracyclines, sometimes administered in BP patients for their anti-inflammatory effects [93,99], and classically regarded as drugs capable of exacerbating psoriasis [100], have been utilized in combination regimens for patients with both psoriasis and BP [10]. Indeed, no apparent psoriasis flare was recorded in patients who received tetracycline-containing regimens for their BP associated with psoriasis [101]. 

The administration of systemic steroids is generally discouraged in psoriasis patients because of the risk of disease flares following their use or withdrawal, although recent evidence has shown that this risk appears to be low in such circumstances [102,103].

A case-control study has demonstrated that adjuvant immunosuppressants were used more frequently in patients with BP and psoriasis as compared with BP patients, probably to avoid the long-term administration of high-dose steroids in psoriatic patients [14].

In their systematic review, Donnelly et al. analyzed treatments used during the acute phase of BP in patients with concurrent psoriasis [10]. Spontaneous recovery was obtained in 3.6% of cases, topical corticosteroids alone were successfully used in 12%, systemic monotherapy gave satisfactory results in 42%, whereas combination therapy (mostly with oral steroids, topical steroids, tetracyclines, and/or methotrexate) was required in 37% of cases. 

Topical corticosteroids were used in addition to systemic therapies in the majority of cases [101]. 

Concerning systemic medications, oral corticosteroids were the most commonly used agents, either alone or in combination regimens, but they were also associated with the majority of negative outcomes, including exacerbation of psoriasis and/or BP when the dose was decreased or treatment was stopped [10,101].

Methotrexate was the second most used systemic medication and proved to be safe and effective for both disorders in many cases. It was also given in association with topical steroids [22,27,72,85,104,105,106], in conjunction with oral and topical corticosteroids [107], or with compound glycyrrhizin in a patient with BP and psoriatic erythroderma [19]. Methotrexate was also utilized, after gradual tapering of cyclosporine [75] or after tapering and discontinuation of systemic steroids [108,109].

Erythromycin was rarely used as it provided no clinical benefits [110]. Tetracycline or doxycycline and niacinamide, generally with topical steroids and sometimes with systemic steroids, were given in a few patients with variable results [30,81,109,110,111,112]. 

Other treatments reported as successful in individual cases include dapsone [110]; cyclosporine [113]; mycophenolate mofetil [114]; acitretin, sometimes associated with topical steroids [23,115,116]; azathioprine [112]; fumaric acids [64]; ustekinumab [85]; ixekizumab [117,118]; etanercept alone [80], combined with an initial short course of prednisone [83], or with subsequent use of acitretin to manage a pustular rash [82].

Other authors noted the inefficacy of mycophenolate mofetil [83] or oral prednisone in addition to dapsone [109].

Among the combination regimens used with satisfactory results, there are cyclosporine with systemic steroids [16,25,80,119]; dapsone and systemic steroids [64,80], along with topical steroids [120]; azathioprine and acitretin [18]; azathioprine and tetracycline [112]; azathioprine and systemic steroids [112,121]; rituximab, plus low-dose methotrexate and a short course of oral prednisolone [122]; rituximab, followed by etanercept [81]; secukinumab, with an initial use of low-dose prednisolone [123]; minocycline and suplatast tosilate [21]; prednisolone, cyclosporine, and dapsone, followed by methotrexate [124]. 

In a patient with BP, psoriasis, and macroglobulinemia, psoriatic lesions improved and bullous lesions disappeared after seven cycles of chemotherapy with dexamethasone, rituximab, and cyclophosphamide for macroglobulinemia [32].

Interestingly, secukinumab resulted in a reduction of anti-BP180 autoantibodies NC16A in a patient with psoriasis and BP, in whom BP was already well-controlled with low-dose prednisolone [125].

Sulfasalazine, acitretin, and topical steroids controlled both psoriasis and BP in a patient with HIV-negative Kaposi’s sarcoma treated with ipilimumab and nivolumab [126].

A recent report has documented the efficacy of the Janus kinase inhibitor baricitinib, in association with a topical steroid, in a case of aggressive BP concurrent with plaque psoriasis [127].

Reported treatments for psoriasis and concurrent BP, other than topical and systemic corticosteroids, are summarized in Table 1.

Hsieh et al., in their comprehensive review regarding the management of BP and psoriasis [101], concluded that corticosteroids and methotrexate were the most popular therapeutic options for more severe cases, followed by azathioprine and cyclosporine, recommending that the dosage should be gradually tapered. Methotrexate, cyclosporine, and azathioprine seemed to be efficacious in both diseases, but methotrexate appeared to be associated with a higher risk of BP and psoriasis aggravation during dose reduction. 

In conclusion, psoriasis with comorbid BP represents a fascinating model of the complexity of the cutaneous inflammatory networks. Although the “epitope spreading” hypothesis remains the most convincing, further research is required to unravel the pathophysiology of this peculiar scenario and to define appropriate therapeutic strategies.

## Figures and Tables

**Table 1:** Main reported treatments for psoriasis and concurrent BP, other than topical and systemic corticosteroids, used as monotherapy or in combination regimens.

Classes	Drugs	References
**Immunomodulating/Immunosuppressant agents**	Methotrexate	[17,19,22,27,72,75,85,104,105,106,107,108,109,118,122,124]
	Cyclosporine	[16,25,75,80,113,119,124]
	Mycophenolate mofetil	[83,114]
	Azathioprine	[18,92,112,121]
	Fumaric acids	[64]
	Suplatast tosilate	[21]
	Baricitinib	[127]
	Cyclophosphamide	[32] *
**Agents with antinflammatory properties**	Erythromycin	[110]
	Tetracycline	[30,81,109,110,112]
	Doxycycline	[111]
	Minocycline	[21]
	Dapsone	[64,80,109,110,120,124]
	Sulfasalazine	[126]
	Nicotinamide	[109,110,111,112]
	Compound glycyrrhizin	[19,126]
**Retinoids**	Acitretin	[18,23,115,116,126]
**Biologics**		
Anti-TNF-alpha	Etanercept	[80,81,82,83]
Anti-IL-12/23	Ustekinumab	[85,91]
Anti-IL-17	Secukinumab	[123,125]
	Ixekizumab	[117,118]
Anti-CD20	Rituximab	[32,81,122] *

BP: bullous pemphigoid; IL: interleukin; TNF: tumor necrosis factor; * administered for the treatment of macroglobulinemia.

## Data Availability

Data sharing is not applicable to this article, as no new data were created or analyzed in this study.
